# The Preparation of the Patient

**Published:** 1920-07-10

**Authors:** 


### PLASTER WORK IN GENERAL PRACTICE.

### II. The Preparation of the Patient.

Before applying any plaster work to the body of the patient there are certain essential preliminaries which should be carefully attended to. For the sake of convenience in description we may group them under three headings, as follows:

I. Measurements.
II. Preparing the site.
III. Ensuring the comfort of the patient while the plaster remains in position.

Nos. II. and III. are really one, but may be separately considered.

I. *Measurements.*—No efficient plaster work can be produced without careful and painstaking preliminary measurements. These are required not only to ensure that the plaster work will fit, but also to allow for any subsequent shrinkage or expansion. Measurements can be taken either with a straight rule, graduated according to the metric system, or with a flexible tape measure. It is well to have both instruments available and in addition to have a bootmaker's adjustable measure for taking measurements of the sole of the foot. Properly to use this latter instrument requires some knowledge of its potentialities, and the practitioner is recommended to pay a visit to the nearest expert bootmaker and to watch how the measure is used, especially when lateral measurements are required. The solid straight rule measure is most serviceable for measuring fixed distances, when a maximum limit is wanted, as in hip joint or arm plaster work; the flexible measure is available for all measurements, but should be most carefully used as mistakes can very easily be made with it. Never use a steel tape measure; its smooth edges are apt to cut the skin, and for fine work it is unreliable owing to the expansion of the metal. Take care that the marks on the measures are clearly legible and that these very useful instruments are not impaired by warping or shrinkage. In addition provide yourself with a good pair of carpenter's callipers, and with an extra large pair for shoulder, chest, and pelvic measurements. I have found a Martin's adjustable pelvimeter the most suitable piece of apparatus for chest measurements, but an ordinary large pair of builder's callipers will answer well. A flexible cyrtometer is sometimes very useful when making plaster corsets with celluloid stiffening, and comes in handy for hip-joint work.

### The Art of Measuring.

When applying the measure be careful of your anatomical details. Select certain fixed points from which to start your measurements and end your measurement at another fixed point. It does not much matter which points you choose, but take care that they are accurately defined. Thus, when measuring the lower leg, if you select the lower edge of the patella, see that the leg is not unduly flexed, and always compare it with the other leg. Bear in mind the anatomical differences in the various stages of growth, especially when you measure children. When you measure the whole lower limb, see that the pelvis is straight and that the two crests of the ilium are on a level with one another. When measuring the neck for a neck corset, in cases of torticollis, see that you make proper allowance for the deviation that exists. Write down your findings at once; do not trust to your memory for such important details. If possible plot out your measurements on cardboard or paper. Better still, secure permanent impressions by using perchloride paper; these are especially useful in cases of scoliosis and flat foot. Always allow for growth of the tissues below the plaster when the latter has been applied and for shrinkage of the splint or corset in the process of drying. The extent of such allowance must be determined by individual circumstances in each case, and no fixed rule can be laid down, but as an average it may be stated that you should allow a quarter of an inch as a maximum over soft parts and an eighth of an inch over bony parts, normally. Where there is pathological swelling these maxima may have to be much increased.

II. *Preparing the Site.*—Whenever you apply plaster work, be careful that the tissues are adequately protected. All bony points must be padded by means of soft material, not necessarily heavily—one layer of cotton wadding is usually sufficient. Superficial structures must be carefully safeguarded against unnecessary pressure. Pay particular attention to junctional points—the elbow, knee, groin, sacro-iliac articulations, costal margins, base of the neck, and axillae. Pad these well, but evenly and with proper attention to the lie of the folds of the skin. Bear in mind that these junctional points are generally better supplied with protection in the shape of sudoriferous glands, and that they are apt to get wet with perspiration, and should, therefore, be dried carefully and the padding so adjusted that no soakage of sweat can accumulate. Be also careful at the wrist and ankle joints, and when you are over the large tendons. A little attention to anatomical details will safeguard you against glaring mistakes, but always bear in mind that you may have to do with abnormal anatomical structures, and make proper allowances for these. I may instance a case in which the peroneal artery was absent and the anterior tibial was the principal source of blood supply to the foot; this fact was not known before a plaster was applied and an unfortunate overcorrection of the foot in plaster led to disastrous results. Especially in over-correction work must you be careful of these details, for a mistake once made is not easily corrected later on.

The next point is to make the foundation of the plaster work as uniformly smooth as possible. This may be variously secured; by means of thin under-

The previous article appeared June 12, p. 271.

**Plaster Work**—(*continued*).

clothing applied to the trunk or limbs, or by careful smooth bandaging with thin flannel or calico. You want as few eminences as possible; all you really need are one or two fixed points for your work to bear upon, and these you must deal with accordingly. Thirdly, give proper attention to the preparation of the patient's skin. Carefully shave it with a safety razor after a liberal lathering; do not neglect this, even with young children. For some reason the growth of hair and down on a limb encased in plaster is very active, much more so, apparently, than on a skin uncovered by plaster, while in addition in some diseases in which plaster is particularly suitable as a medium for treatment, hair and down growth is equally rich. Use a safety razor for preference; it is more convenient, gives less chance of injuring the skin, and is far more agreeble to the patient than the ordinary razor. Having done the shaving, carefully dry the limb, and then rub in with oil or with some toilet powder. Never use Fuller's earth; my own rule is to employ sterile powder, which is a mixture of chalk, zinc oxide, and a little bismuth carbonate to which a few drops of oil of wintergreen have been added; this is a smooth, pleasant preparation which never irritates the most sensitive skin. All open sores sinuses, or excoriations must be properly protected, and in many cases outlets for dressing purposes must be left in the plaster. Do not attempt to "harden" the skin by applying Eau de Cologne or alcohol; this custom is still in vogue as a reputed preventative against bed sores, but it is doubtful if it is of real utility; cleanliness and proper prevention of undue pressure are all that is required to prevent ulceration at the "bearing points."

III. *Ensuring the Patient's After-Comfort.*—Attention to the little details enumerated above is half the battle. You will find, however, that a great deal still remains to be done to ensure the patient's comfort once the splint or corset has been fixed. Itching, discomfort, and pain may be complained of. These are the results of (*a*) bad preparation, (*b*) irritation by sweat under the plaster, and (*c*) insufficient protection of the tissues beneath the plaster. They have all been dealt with in the foregoing paragraphs. If your plaster has been properly "built" to adapt itself to the structures on which it lies, you may yet find that the irritation from natural secretions will give some trouble; in such cases it is best to remove the splint and to apply drying powders to the skin before re-application of the plaster. A foundation of flannel often gives trouble through itching; in general, therefore, use thin calico for foundations, especially when working with children. Pain not due to the plaster itself must be treated on special lines. In young children who are restless and irritable, an injection of morphia immediately after the application of the plaster is sometimes necessary, especially where an anæsthetic has been used and vomiting is expected as a result. I have never seen any harmful results follow an injection of a quarter ampoule of omnopon to children in these circumstances, and the hint is worth remembering.

Nursing.

Finally, see that the patient's bed is in proper order, and that he gets adequate nursing. When it is an ambulatory case, see that the proper educative treatment is given, and that good food and proper tonic treatment are available. A new splint often gives rise to discomfort, and the patients must be encouraged to bear with it until they get accustomed to the novelty. In the case of children, who readily respond to suggestion, numerous ways of dealing with this trouble will suggest themselves to the practitioner, and, as a matter of fact, adult patients give far more worry when this difficulty is encountered than do children.

